# Neuronal Shot Noise and Brownian 1/f^2^ Behavior in the Local Field Potential

**DOI:** 10.1371/journal.pone.0004338

**Published:** 2009-02-03

**Authors:** Joshua Milstein, Florian Mormann, Itzhak Fried, Christof Koch

**Affiliations:** 1 California Institute of Technology, Pasadena, California, United States of America; 2 Department of Neurosurgery, David Geffen School of Medicine and Semel Institute of Neuroscience and Human Behavior, University of California Los Angeles, Los Angeles, California, United States of America; 3 Functional Neurosurgery Unit, Tel-Aviv Medical Center and Sackler Faculty of Medicine, Tel-Aviv University, Tel-Aviv, Israel; Instituto Cajal - CSIC, Spain

## Abstract

We demonstrate that human electrophysiological recordings of the local field potential (LFP) from intracranial electrodes, acquired from a variety of cerebral regions, show a ubiquitous 1/f^2^ scaling within the power spectrum. We develop a quantitative model that treats the generation of these fields in an analogous way to that of electronic shot noise, and use this model to specifically address the cause of this 1/f^2^ Brownian noise. The model gives way to two analytically tractable solutions, both displaying Brownian noise: 1) uncorrelated cells that display sharp initial activity, whose extracellular fields slowly decay in time and 2) rapidly firing, temporally correlated cells that generate UP-DOWN states.

## Introduction

Power laws appear in a large variety of settings throughout nature and often signify that there is a simple process at the origin of what appears to be a very complex phenomenon. Examples of the variety of settings in which power laws appear are the Gutenberg-Richter law for the size of earthquakes [1], [2], the allometric scaling laws that appear throughout biology [3], and Paretos's law of income distributions [4].

Power laws have also been witnessed within the brain in electroencephalographic (EEG) and magnetoencephalographic (MEG) recordings while studying a wide variety of brain function [5], [6], [7]. The signals recorded outside the skull by these techniques represent the global activity of a large amount of cortical and subcortical tissue and give rise to a range of exponents (for an overview see reference [8]). Much more local measurements of cerebral activity may be recorded by a single microelectrode. While the emphasis of these measurements is usually focused on the spiking activity of single cells within the vicinity of the recording electrode, local field potentials (LFPs), which comprise the much slower background of electrical activity, may also be extracted from the signal. Moreover, previous studies have shown that the local brain dynamics recorded from microelectrodes can be masked in the coarse bulk signal recorded from an intracranial macroelectrode [9]. While it may be assumed that the temporal behavior of EEGs is similar to that of LFPs, this assumption remains to be proven. In fact, connecting local behavior, such as that displayed by single-neuron activity and LFP measurements, to more global measurements such as EEG recordings, is one of the great challenges to our understanding of the brain.

We found that electrophysiological recordings, taken from pharmacologically intractable epilepsy patients with microelectrodes implanted in a variety of cerebral areas, display a surprisingly universal 1/f^2^ power law in the frequency spectrum of LFP activity. To the best of our knowledge, this is the first time such a universal feature of the LFP has been reported in humans.

A 1/f^2^ power spectrum is said to display the statistics of Brownian noise since it has the same scaling exponent as a 1D random walk. However, it is far from clear what the underlying mechanism is that gives rise to these statistics. Various other studies have tried to address the issue of scale free phenomena within the brain by invoking concepts such as self-organized criticality [10], [11] or by imposing frequency dependences on the intercellular medium [12]. While the relevance of the former in explaining these phenomena can be debated [13], recent experiments make the later seem unlikely [14]. In the current paper, we take an alternative approach, that is, we focus on understanding how an extracellular field could give rise to such scale-free phenomena. To this end, we developed a general method for modeling the LFP from what we refer to as neuronal shot noise, which allows one to study the microscopic origin (i.e., single neuron activity) of the power law dependence in the power spectrum. We propose two quite different processes that could both give rise to the observed 1/f^2^ dependence. The first involves the uncorrelated firing of single neurons that display very slow dendro-synaptic decay in the extracellular field which they generate. The second possibility involves the correlated firing of a single neuron that displays either no activity (DOWN state) or very rapid spiking (UP state). We end with a discussion of the UP-DOWN states (UDS) suggested by our model and how they compare to experimentally observed UDS within the cortex.

## Methods

### Experimental Methods

We recorded local field potentials from the cerebral cortex of 10 subjects with pharmacologically intractable epilepsy (4 males; 24–46 years old), implanted with chronic electrodes to localize the seizure focus for possible surgical resection. Electrode locations were based exclusively on clinical criteria and were verified by MRI or by computed tomography co-registered to preoperative MRI. Each electrode probe had nine micro-wires (Platinum/Iridium, 40 µm diameter) protruding from its tip, eight high-impedance recording channels (typically 200–400 kΩ) and one low-impedance reference with stripped insulation. The differential signal from the micro-wires was amplified using a 64-channel Neuralynx system, filtered between 1 and 9000 Hz, and sampled at 28 kHz. We recorded from a total of 684 micro-wires (106 in the frontal lobe, 546 in the temporal lobe, 16 in the parietal lobe, 16 in the occipital lobe). For more technical information, as well as detailed images of the electrodes, we refer the reader to Bragin et al. [15].

Recordings lasted for 10 minutes while subjects were awake and at rest with eyes open. If the subjects' eyes closed, an alpha rhythm appeared as a nested oscillation around 10 Hz on top of the spectrum that impairs the goodness-of-fit for our estimation of the scaling exponent. Since we were primarily concerned with this scaling exponent, we preferred a setting without a dominant rhythm. The recording conditions were stationary over those 10 minutes.

Recordings were typically performed at the beginning of the pre-surgical monitoring, i.e. one day after implantation of the electrodes, but before withdrawal of anti-epileptic medication, so patients were still stable on their normal medication. No additional anesthetics were administered. Moreover, recordings were typically performed several days before the first seizure was recorded during monitoring. Written informed consent was obtained from all human subjects and all studies conformed to the guidelines of the Medical Institutional Review Board at UCLA [16].

**Figure 1:**
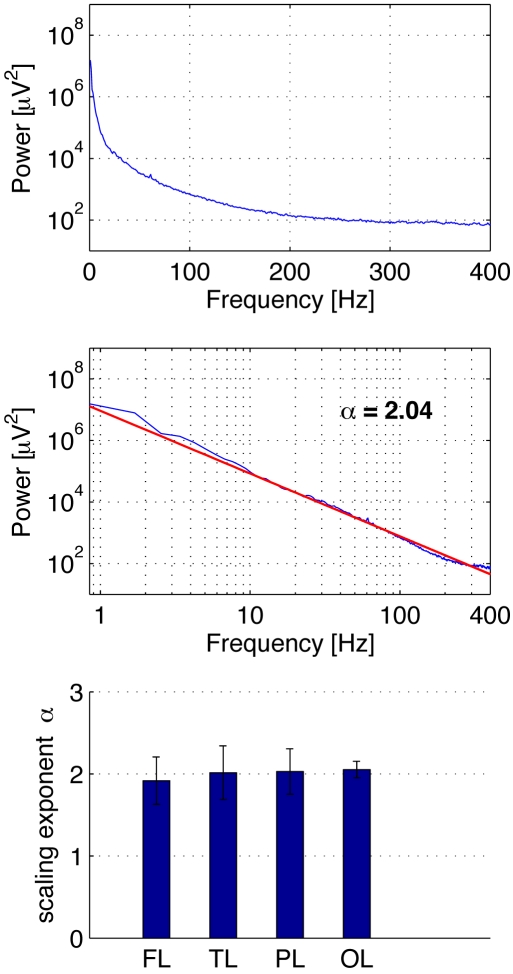
Power law and scaling exponent in local field potentials recorded from the human cerebral cortex. Top: Exemplary power spectrum of local field potentials recorded from a micro-wire in the temporal lobe. Middle: The scaling exponent (here α = 2.04) was determined by a linear least-square fit of the log-log power spectrum. Bottom: Scaling exponents (mean±stand. dev.), averaged across micro-wires for different brain regions. FL: frontal lobe α = 1.92±0.29; TL: temporal lobe α = 2.02±0.33; PL: parietal lobe α = 2.03±0.28; OL: occipital lobe α = 2.05±0.10.

For analysis, the data was down-sampled to 7 kHz using an anti-aliasing filter. The power spectral density was estimated by applying Welch's method to consecutive 5-sec segments and subsequently averaging over the entire 10 min (Fig. 1). The scaling parameter a was determined as the slope of a least-square linear fit of the double-logarithmic power spectrum. To diminish the influence of amplifier roll-off, the linear fit was restricted to a frequency range of 1 to 400 Hz.

### Theoretical Model

**Figure 2:**
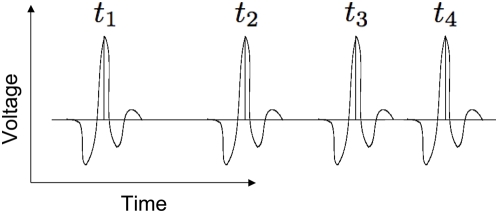
Schematic of Eq. 1 representing neuronal shot noise. A function f_i_(t), representing the extracellular field associated with the i^th^ neuron, occurs at times t_1_,t_2_,… governed by the statistics of μ_i_(t).

The microscopic generation of the Local Field Potential (LFP) may be formulated in a similar way to that of shot noise, originally described by Schottky [17] to account for the noise across an electrical resistor. This may be seen by writing the extracellular potential *V*(*t*) generated by 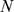 neurons, at a given spatial location within the brain, as follows:
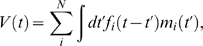
(1)which is exactly how one quantitatively models shot noise. Here the function f_i_(t) accounts for the temporal profile of the extracellular field generated by neuron *i* while μ_i_(t) represents the activity of that neuron and may be explicitly written as
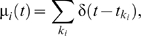
(2)where δ(*t*) is the Dirac delta function. From this definition, we see that the function μ_i_(t) may be thought of as analogous to the spike train with firing activity occurring at times t_ki_ for neurons *i* = 1…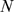. Note, this model does not require that the neurons generate action potentials; it only assumes a stereotyped profile f_i_(t) for the electrical field generated by each neuron which repeats at times t_ki_ (see Fig. 2).

We will assume that the relevant neural activity has reached a steady-state such that the autocorrelation *G*(τ) = 〈*V*(*t*)*V*(*t*+τ)〉 is independent of *t*. By the Wiener-Kinchin theorem, the autocorrelation function *G*(*t*) is related to the power spectrum *S*(ω) by Fourier transformation. From Eq. 1, we may write the power spectrum as
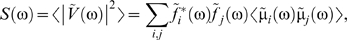
(3)where 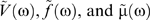 are the Fourier transforms of their respective temporal functions.

To solve for the power spectrum as written in Eq. 4 would require us to know the location of each neuron involved in generating the LFP, the extracellular field produced by each neuron, and the decay of that field through the neuronal medium. While we have carried out such biophysical calculations in the past for single neurons [18], [19], [20], we are here only concerned with understanding the source of the power law behavior of the power spectrum, not in reconstructing the LFP.

The scale invariant nature of *S*(ω) greatly simplifies our problem since it will allow us to neglect many of the biophysical constants that arise from the details mentioned above. To clarify this point, let us assume that the power spectrum S(ω) = C_1_ω^n^, where C_1_ is a constant. We can solve for the coefficient of the power by plotting the log of both sides of this equation, log S(ω) = n·log(ω)+log(C_1_). The power dependence is given by the slope *n* and is unaffected by the constant offset.

## Results

Figure 1 displays the experimental values of the scaling parameters found in our recordings. The scaling parameters where averaged across different micro-wires for four regions of the cerebral cortex along with their standard deviation. The observed values for the scaling exponent±standard deviation are: Frontal Lobe (FL) α = 1.92±0.29, Temporal Lobe (TL) α = 2.02±0.33, Parietal Lobe (PL) α = 2.03±0.28, Occipital Lobe (OL) α = 2.05±0.10. Note that in all four regions the scaling parameter is close to a value of α = 2, indicating a universal scaling behavior of local electrical brain activity.

Using the simplified model discussed in the methods section above, we now focus on searching for solutions of Eq. 3 that have a 1/ω^2^ functional dependence. In general, it is quite difficult to evaluate Eq. 3; however, there are two limits that allow a simple, analytical solution. We now discuss these two cases.

### Case I: Slow Dendro-Synaptic Decay

The simplest case to consider is that the spiking statistics are independent between neurons, and that the spiking of each neuron is an independent Poisson process [21]. In this case
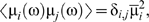
(4)where 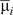 is the average firing rate of the neuron *i* and δ_i,j_ is the Kronecker delta function.

We can now ask when the power spectrum satisfies
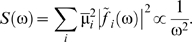
(5)


The solution requires 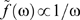, whose Fourier transform is a Heaviside step function f_i_(t)∝Θ(t). This answer is a bit unrealistic since it implies that the field generated by the cell does not decay with time. A more realistic solution would be to assume a form such as
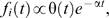
(6)which has Fourier transform 1/(α+*i*ω). In the limit of slow decay, α≪1, a neuron with an extracellular field of this form, firing with Poisson statistics, would give rise to Brownian noise in the LFP.

In this case, the 1/f^2^ behavior originates from the steep onset of the extracellular field. The rise time of an action potential may occur within a fraction of a millisecond, which could account for a sharp onset, while the decay of the dendro-synaptic extracellular field may last for as long as a second [22]. The functional form of the decay does not affect these results, so long as the cell takes much longer to return to baseline than it took to spike.

### Case II: UP-DOWN States

The second case that we consider is the limit of a sharply peaked extracellular field. In this case, we may treat 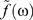 as a constant 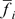, and we will assume that the activity of different neurons is either uncorrelated (〈μ_i_μ_j_〉∝δ_I,j_) or synchronous (〈μ_i_μ_j_〉∝1). The spike timing of a single neuron, however, may show a temporal correlational structure. These assumptions lead to a power spectrum
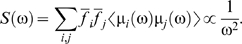
(7)


Since all the frequency behavior is contained within the statistics of μ_i_, and we are assuming that all cells are active with the same statistics, we need to look for a sequence of spikes that have individual spike timing correlations of the form
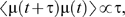
(8)since linear time correlations are consistent with 1/ω^2^ frequency correlations. This is the same linear in τ scaling as that of a 1D random walk and is at the origin of the term Brownian noise.

Since μ(*t*) is analogous to the spike train of each neuron, we need to formulate a binary sequence that shares the correlational structure of a random walk. A simple way to generate a binary sequence representing white noise is to pick a random number at each timestep and then apply a threshold such that all values above the threshold are set to one, and all below to zero. Brownian noise may be created by integrating a white noise signal. However, it is not obvious how to apply a similar thresholding procedure to convert the resulting analog signal into a digital one. For instance, one may limit the random walk to positive numbers and set a threshold. A binary sequence is generated by adding a 0 at each timestep that the walker remains below the threshold. If the threshold is reached, a 1 is added to the binary sequence, the walker is reset to the origin, and the process continues. Unfortunately, the resetting procedure clears the memory of the random walker, and we again generate a flat, white noise spectrum.

**Figure 3:**
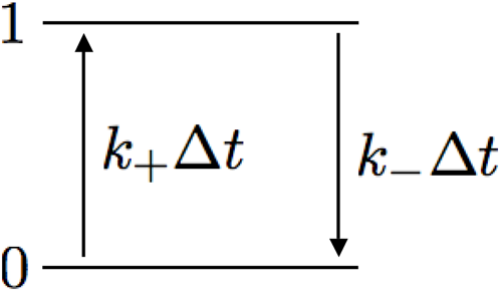
Schematic of a telegraph process used to generate a binary sequence of spike times. At each timestep the binary variable makes the transition from 0→1 (resp. 1→0) with probability k_+_Δt (resp. k_−_Δt).

An alternative procedure that will generate Brownian noise is given by setting up a telegraph process (see Fig. 3) [23]. In this case a binary sequence is generated by constructing a two-state system, (0 and 1) where, at each timestep, the probability of making the transition 0→1 (1→0) is given by k_+_Δt (k_−_Δt). The autocorrelation function for such a process may be explicitly written as
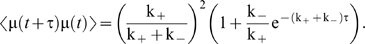
(9)


In the limit of equal transition rates, k_+_ = k_−_, and low probability of making a transition, (k_+_+k_−_)τ≪1, Eq. 9 reduces to
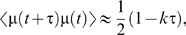
(10)which has the desired linear in τ statistics of a random walk.

**Figure 4:**
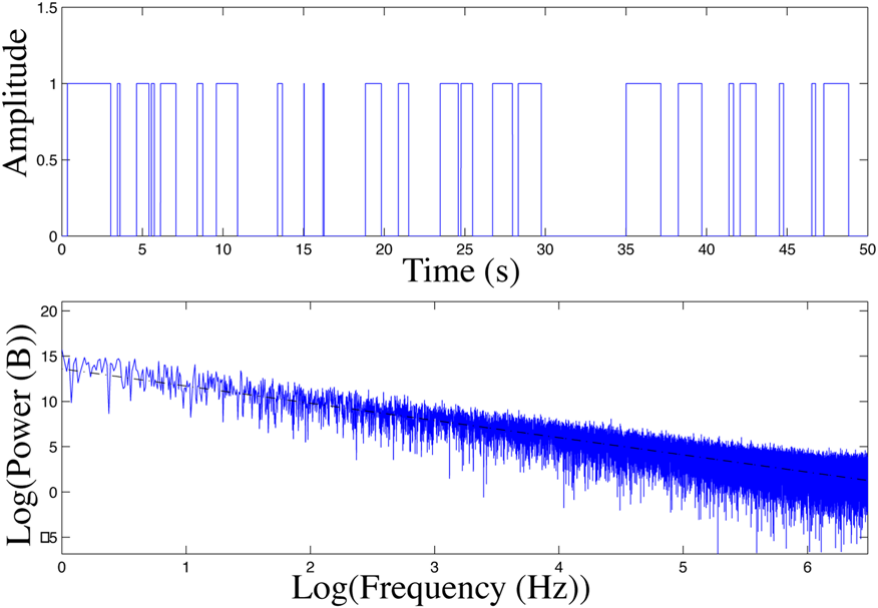
Modeling of UP-DOWN states by a telegraph process. Top: Binary sequence generated from a telegraph process. Bottom: Power spectrum of the binary sequence confirming the 1/f^2^ behavior. The slope of the dashed line is −2.

Figure 4 displays a binary sequence generated by a telegraph process and the 1/f^2^ dependence of its power spectrum. The telegraph process gives rise to periods of sustained, rapid activity followed by intervals of inactivity. This results in collective oscillations that display a much lower frequency than the rapid firing witnessed during depolarization. The result is a pattern of behavior reminiscent of UP-DOWN states common in cortex [24].

## Discussion

Studies of the LFP and single neuron spiking activity, combined with current source-density analysis, suggest that LFPs are primarily the result of dendritic activity distributed over a large region of the cortex. LFPs are therefore believed to provide a measure of the local processing and input to a given region of the brain [25], [26].

We developed a very simple model to explain our key experimental finding, a 1/f^2^ decay in the local field potential recorded from individual microelectrodes implanted into human cortex. In particular, we showed two examples of how biologically realistic, single neuron activity, parameterized by the temporal shape of the resulting extracellular fields and the statistics of cellular activity, can give rise to power law behavior within the LFP.

In Case I , we showed how a population of cells, each displaying a sharp onset of activity and a much slower decay of the extracellular field, could give rise to a Brownian power spectrum. The time course of dendritic activity is often much longer than that of an action potential. This is in line with the above statement concerning the origin of the LFP. However, the sharpness of the temporal onset of activity is what gives rise to the power-law behavior. One mechanism that might account for this result would be the rapid initiation of an action potential, followed by slow dendro-synaptic decay.

Of course, this model not only assumes that the spiking statistics of each neuron is Poisson, but that there are no correlations among neurons. It is not uncommon to find the firing rate of single neurons uncorrelated with the averaged behavior of the local population; however, this is not always the case [27].

In Case II, we explored the opposite extreme from Case I, that of rapidly firing, single neurons with linear temporal correlations. This behavior is similar in nature to so-called UP-DOWN states seen in cortical neurons. During periods of sleep, quiet awake behavior, or under a variety of anesthetics, spontaneous activity of neocortical neurons display slow 0.1 to 2 Hz oscillations called UP-DOWN states (UDS). These states appear to be characteristic of slow-wave sleep [28], [29], [30] and are thought to be involved in the consolidation of long-term memories and in learning. The UDS of cortical pyramidal neurons are highly synchronized and may be clearly seen in LFP recordings of the cortex. The UP states are characterized by a sustained depolarization that leads to rapid, 20–70 Hz spiking activity while the DOWN states show periods of hyper-polarized inactivity.

It should be pointed out that our recordings were performed in the awake resting state in the human cortex, whereas UDS and ultra-slow oscillations have been described only in states of low vigilance such as slow-wave sleep and anesthesia in animal studies. It is therefore unlikely that the power law scaling behavior observed in our recordings would be caused exclusively by the mechanisms illustrated in Case II. Nevertheless, it is encouraging that this extreme analytical case of a 1/f^2^ power law scaling gives rise to phenomena that are actually observed in mammalian brains.

The true origin of the 1/f^2^ behavior probably lies somewhere in-between the two limiting cases we have considered here. Unfortunately, an analytic evaluation of Eq. 1 when there is explicit time dependence in both the extracellular field (f_i_(t)) and the firing statistics (μ_i_(t)) is, in general, difficult. However, for a known set of f_i_(t) and μ_i_(t), a numerical evaluation of Eq. 1 is straightforward. This formalism should, therefore, serve as a starting point in modeling power-law dependencies in the power spectrum of the LFP and in connecting this property to the underlying single neuron activity.
